# Association between urinary albumin creatinine ratio and cardiovascular disease

**DOI:** 10.1371/journal.pone.0283083

**Published:** 2023-03-21

**Authors:** Yoo Jin Kim, Sang Won Hwang, Taesic Lee, Jun Young Lee, Young Uh

**Affiliations:** 1 Division of Nephrology, Department of Internal Medicine, Yonsei University Wonju College of Medicine, Wonju, Korea; 2 Artificial Intelligence Bigdata Medical Center, Yonsei University Wonju College of Medicine, Wonju, Korea; 3 Department of Family Medicine, Yonsei University Wonju College of Medicine, Wonju, Korea; 4 The Study of Obesity and Metabolic Syndrome, KAFM, Korea; 5 Department of Laboratory Medicine, Yonsei University Wonju College of Medicine, Wonju, Korea; Bolu Abant İzzet Baysal University: Bolu Abant Izzet Baysal Universitesi, TURKEY

## Abstract

**Introduction:**

The association between microalbuminuria and cardiovascular disease (CVD) is accumulating in various patient populations. However, when stratified by sex, the relationship between microalbuminuria and CVD remains unclear.

**Method:**

We obtained data from the 2011–2014 and 2019–2020 Korea National Health and Nutrition Examination Survey (KNHANES). Microalbuminuria was measured based on spot urine albumin-creatinine ratio (UACR). The Framingham risk score (FRS) model was implemented to evaluate the CVD risk. Linear and logistic regression models were used to identify the associations of microalbuminuria status with cardiometabolic predictors and CVD status determined by the FRS score.

**Results:**

Among 19,340 representative Korean participants, the (UACR) in Korean women and men with history of CVD was higher than in those without history of CVD. Among patients without history of CVD, multivariate regression analysis showed that a high UACR was related to older age, lower high-density lipoprotein cholesterol level, higher total cholesterol level, higher systolic blood pressure, higher prevalence of current smoking, higher prevalence of diabetes, and higher anti-hypertensive medication use in both women and men. The UACR showed a positive linear correlation with the Framingham risk score in both women and men.

**Conclusion:**

The presence of microalbuminuria was significantly associated with the cardiometabolic risk factors and the increased risk of CVD evaluated by FRS model in both women and men in a nationally representative sample of Korea.

## Introduction

Cumulative evidence indicates that albuminuria is associated with increased risk of cardiovascular diseases (CVDs) [1, 2]. The Heart Outcomes Prevention Evaluation (HOPE) study concluded that any degree of albuminuria (e.g., microalbuminuria) is a risk factor for cardiovascular (CV) events; particularly, a 0.4-mg/mmol increase in the albumin-creatinine ratio (ACR) was related to a 5.9% increased risk for CV events [1]. The Prevention of Renal and Vascular End Stage Disease (PREVEND) study, conducted among inhabitants of the city of Groningen (the Netherlands), reported that a 2-fold increase in albuminuria was associated with a 1.29 and 1.12-times increased risk for CV mortality and non-CV mortality, respectively [2]. The Prevention of Events with an ACE inhibitor (PEACE) trial showed that albuminuria, even at low levels within the normal range, is an independent predictor of CV mortality (hazard ratio per log ACR:1.74) [3]. This evidence comes from cohort studies not only from individuals at high risk of CVDs (patients with diabetes, hypertension, older adults, or stable coronary artery disease), but also from the general population [2–5]. Several studies have shown this association, even in patients with microalbuminuria [1, 6]. However, most studies pertain to sex-adjusted CV risk and no study has revealed sex-specific CV risk.

The Korea National Health and Nutrition Examination Survey (KNHANES) is a nationwide cohort in Korea that has collected urine albumin levels of participants since 2011. Initial studies that used the urinary albumin data from KNHAENS focused on identifying the risk factors related to albuminuria and/or microalbuminuria [7, 8]. Eventually, Korean nephrologists analyzed the 2011–2013 KNHANES, and reported clinical predictors related to albuminuria and/or chronic kidney disease (CKD) [9]. The accumulation of urine albumin data in KNHANES has diversified research topics. Body composition-related health problems have been studied as candidate association factors with albuminuria. Several studies using the KNHANES have reported the independent association between sarcopenia (also referred to as low skeletal muscle mass status) and albuminuria [10, 11]. A study analyzing urine albumin and body composition data in the KNHANES demonstrated that urine albumin level is related to bone mineral density of total hip in postmenopausal women [12].

Meanwhile, only a few studies analyzing KNHANES data have pinpointed the association between albuminuria and CVD [13]. Ahn et al. [13] obtained urine albumin data from the 2011–2013 KNHANES, and demonstrated that albuminuria could reflect CVD risk as measured by the Framingham risk score (FRS) [14]. However, this study only reported findings among postmenopausal women without diabetes [14]. Taken together, the current study aimed to evaluate the relationship between urine albumin-creatinine ratio (UACR) and CVD according to sex.

## Methods

### Ethics statement

All the participants enrolled in the KNHANES signed an informed consent form. The KNHANES data and their analyses in the present study were performed in compliance with the Declaration of Helsinki. The present study protocol was approved by the Institutional Review Board of Wonju Severance Christian Hospital (IRB No. CR321375).

### Study population

This study analyzed data obtained from the 2011–2014 and 2019–2020 KNHANES. The KNHANES is conducted annually by the Division of Chronic Disease Surveillance of the Korea Centers for Disease Control and Prevention in the Ministry of Health and Welfare to assess and monitor the general and medical health and nutrition status in South Korea [15, 16]. The KNHANES includes three main components, a health interview, health examination, and nutrition survey. The KNHANES implements a complex, multi-stage probability sample design to obtain nationally representative data [15, 16]. The KNHANES is publicly available data (https://knhanes.kdca.go.kr/knhanes/sub03/sub03_02_05.do). Among 47,613 participants in the KNHANES, we excluded those aged under 40 years (n = 24,982), and those with missing information on demographics, lifestyle, medical, anthropometric, and laboratory variables (n = 3,291). After the exclusion, a total of 19,340 participants were analyzed.

### Measurement of urine albumin and creatinine

The gold standard for measuring urine albumin excretion is 24-h urine collection. However, the National Kidney Foundation recommends the use of spot urine albumin-creatinine ratio to detect microalbuminuria, which is more convenient and accurate than 24-h urine collection. However, the cutoff value to diagnose microalbuminuria is different across different races and sex [17]. Therefore, we estimated sex-specific UACR and CV risk using data from the KNHANES.

### Covariates

Old age, diet (low intake of vegetables, fruits, and whole grain; and high intake of processed red meats, refined carbohydrates, and sweetened beverages), low or irregular physical activity, diabetes, and tobacco use are widely known risk factors for atherosclerotic CVD (ASCVD) or its related mortality [18]. Moreover, other factors, such as serum lipid profiles, air pollution, and genetic factors, are related to ASCVD [19–21].

From representative models [22, 23], we determined seven predictors: age, total cholesterol (TC), high-density lipoprotein cholesterol (HDL-C), systolic blood pressure (SBP), antihypertensive medication (AHM), current smoking (CS), and diabetes as covariates.

### Measurement of CVD risk score based on Framingham risk score

CVD risk score was measured using the FRS model [22]. The FRS model was established based on the Cox proportional hazards model which is widely used in the medical field [24]. The Cox model includes a linear unit and a non-linear unit (termed to survival function [22, 24]). The FRS includes an interaction term AHM×SBP, for which, the coefficient is 0.06106 (2.82263–2.76157) in women and 0.06578 (1.99881–1.93303) in men. The equation for calculating the FRS is as follows.

Linear predictor (LP)_women_ = ln(age)×2.32888 + ln(TC) ×1.20904 + ln(HDL-C) ×(–0.70833) + ln(SBP)×2.76157 + ln(SBP)×AHM (0:no; 1: yes)×(2.82263–2.76157) + current smoking status × 0.52873 + diabetes×0.69154–26.1931

FRS_women_ [22] = 1–0.95012^exp(LPwomen)^

FRS_men_ [22] = ln(age)×3.06117+ ln(TC) ×1.12370 + ln(HDL-C) ×(–0.93263) + ln(SBP)×1.93303 + ln(SBP)×AHM (0:no; 1: yes)×(1.99881–1.93303) + current smoking status × 0.65451 + diabetes×0.57367–23.9802

FRS_men_ [22] = 1–0.88936^exp(Lpmen)^

### Statistics

R language (version 4.0.1) [25] was implemented to reconstruct and preprocess the dataset and perform statistical analysis. Continuous variables, such as demographics (e.g., age) and laboratory values, were analyzed using ANOVAR. For categorical variables, the chi-square test was utilized. To evaluate the linear trends of categorical or continuous variables based on UACR tertile, we determined the median UACR levels of each tertile group as continuous variables when using the Chi-square test and one-way ANOVA.

To estimate the total population that the data would represent, we employed the sampling weights determined by the data constructors. After adopting the weight values, we analyzed the association between UACR and cardiometabolic risk factors included in the FRS equation. Multivariate linear or logistic regressions were performed using the following equation: *cardiometabolic risk factors* (dependent variable) *~ UACR* (independent variable) *+ covariates*. A *p*-value of < 0.05 was determined to be statistically significant.

## Results

The distribution of urine albumin (S1 Fig) among Korean women did not show abnormal distribution; instead, the distribution skewed left, indicating the existence of several outliers of urine albumin levels. After inclusion of cases with urine albumin less than 10 mg/dL, the patterns still did not show normal distribution; instead, they showed gamma or log-normal distributions. In contrast, urine creatinine levels in Korean women slightly skewed to the left, but generally followed a normal distribution. The distribution of UACR values in Korean men was similar to that of their urine albumin levels. These findings were consistent among Korean men (S2 Fig).

**Fig 1:**
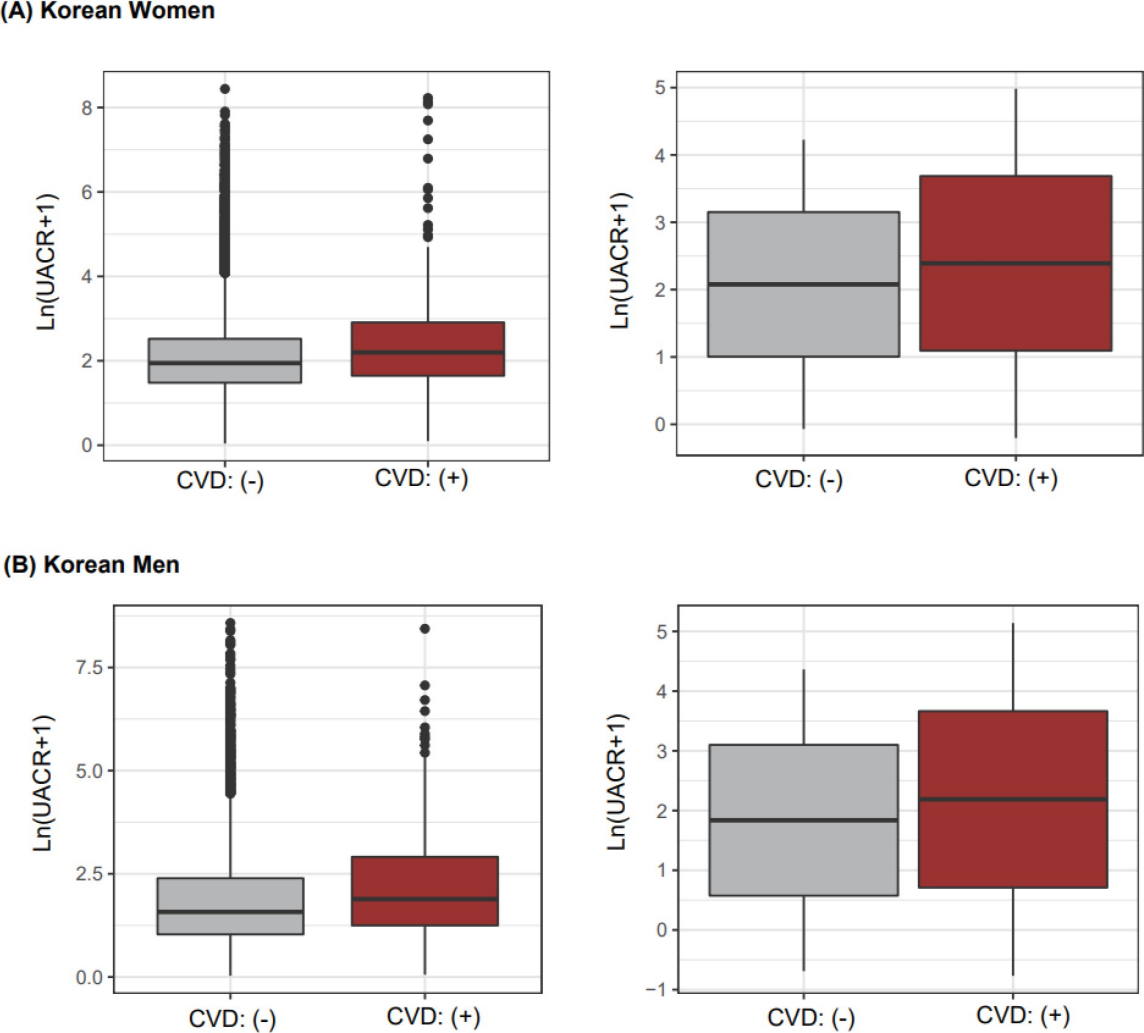
UACRs according to CVD status in Korean women (A) and men (B). Left side boxplots (grey and brown colored boxes) indicate median-based summary statistics; specifically, the middle, upper, and lower lines describe median, 75, and 25 percentile values, respectively. Right side boxplots indicate mean-based summary statistics, in which the middle, upper, and lower lines illustrate mean, one standard deviation values, respectively.

When comparing UACR levels according to the CVD status obtained from questionnaires, both mean and median values of UACR among Korean women were higher in the CVD group than in the non-CVD group (median [non-CVD/CVD groups] = 5.99/8 mg/g; mean = 20.8/60.8 mg/g, Fig 1A). However, in the non-CVD group, many outliers had extremely high UACRs because most participants were in the non-CVD group (Fig 1A). These results were also exhibited among Korean men because the prevalence of CVD was low in both Korean men and women (Fig 1A). Among the self-reported responses, we found that the UACR was high in CVD status; therefore, as the next step, we evaluated the association between UACR, and CVD status measured by FRS score after excluding CVD patients.

Different general characteristics were shown among Korean women according to increasing UACR: older age, higher SBP, higher TC, lower HDL-C, greater AHM use, higher prevalence in diabetes, and higher levels of FRS. For Korean men, most risk factors, except for the serum levels of TC, exhibited similar bio-signatures compared to those of Korean women (Table 1). However, as the UACR increased from tertile 1 to tertile 3, the number of current smokers did not show any significant difference between men and women. Note that all participants analyzed in Table 1 were not diagnosed with CVD.

**Table 1:** Sex-specific characteristics according to UACR tertile.

	Korean women			
Variable	T1	T2	T3	p-value
Unweighted participants, n	2,325	2,324	2,332	
Age, years	55.3 ± 0.22	57.6 ± 0.23	62.5 ± 0.23	<0.001
Systolic BP, mmHg	116.1 ± 0.32	119.6 ± 0.35	128.8 ± 0.35	<0.001
Antihypertensive medication, n	424 (18.2)	596 (25.6)	982 (42.1)	<0.001
Diabetes, n	120 (5.2)	180 (7.7)	374 (16)	<0.001
Current smoker, n	79 (3.4)	84 (3.6)	83 (3.6)	0.917
Total cholesterol, mg/dL	197.7 ± 0.74	197.1 ± 0.75	199.3 ± 0.75	<0.001
HDL-cholesterol, mg/dL	52.9 ± 0.25	52.5 ± 0.25	50.3 ± 0.25	<0.001
Urine albumin, mg/dL	0.2 ± 0	0.7 ± 0.01	4.7 ± 0.01	<0.001
Urine creatinine, mg/dL	101.6 ± 1.15	129.2 ± 1.45	115.5 ± 1.44	<0.001
UACR, mg/g	1.5 ± 0.02	5.1 ± 0.03	52.4 ± 0.03	<0.001
FRS	0.065 ± 0.001	0.079 ± 0.001	0.126 ± 0.001	<0.001
	Korean men			
Unweighted participants, n	1,669	1,669	1,675	
Age, years	56.6 ± 0.27	57.9 ± 0.27	62.5 ± 0.27	<0.001
Systolic BP, mmHg	119.3 ± 0.36	121.8 ± 0.38	128.8 ± 0.38	<0.001
Antihypertensive medication, n	323 (19.4)	403 (24.1)	675 (40.3)	<0.001
Diabetes, n	98 (5.9)	135 (8.1)	414 (24.7)	<0.001
Current smoker, n	556 (33.3)	617 (37)	594 (35.5)	0.085
Total cholesterol, mg/dL	189.5 ± 0.84	190.1 ± 0.81	186.4 ± 0.81	<0.001
HDL-cholesterol, mg/dL	47.4 ± 0.28	47.3 ± 0.28	46.3 ± 0.28	<0.001
Urine albumin, mg/dL	0.2 ± 0	0.7 ± 0.01	9.9 ± 0.01	<0.001
Urine creatinine, mg/dL	142 ± 1.66	176.2 ± 1.95	155.3 ± 1.94	0.999
UACR, mg/g	1.1 ± 0.02	4 ± 0.03	80.9 ± 0.03	<0.001
FRS	0.161 ± 0.003	0.185 ± 0.003	0.272 ± 0.003	<0.001

Continuous and categorical variables are presented as mean ± standard error and number (percent), respectively. *p*-values for the association of continuous variables with UACR were calculated using ANOVA and those for categorical variables were calculated using the Chi-square test.

Abbreviations: UACR, urinary albumin-creatinine ratio; BP, blood pressure; HDL, high-density lipoprotein; FRS, Framingham risk score.

**Fig 2:**
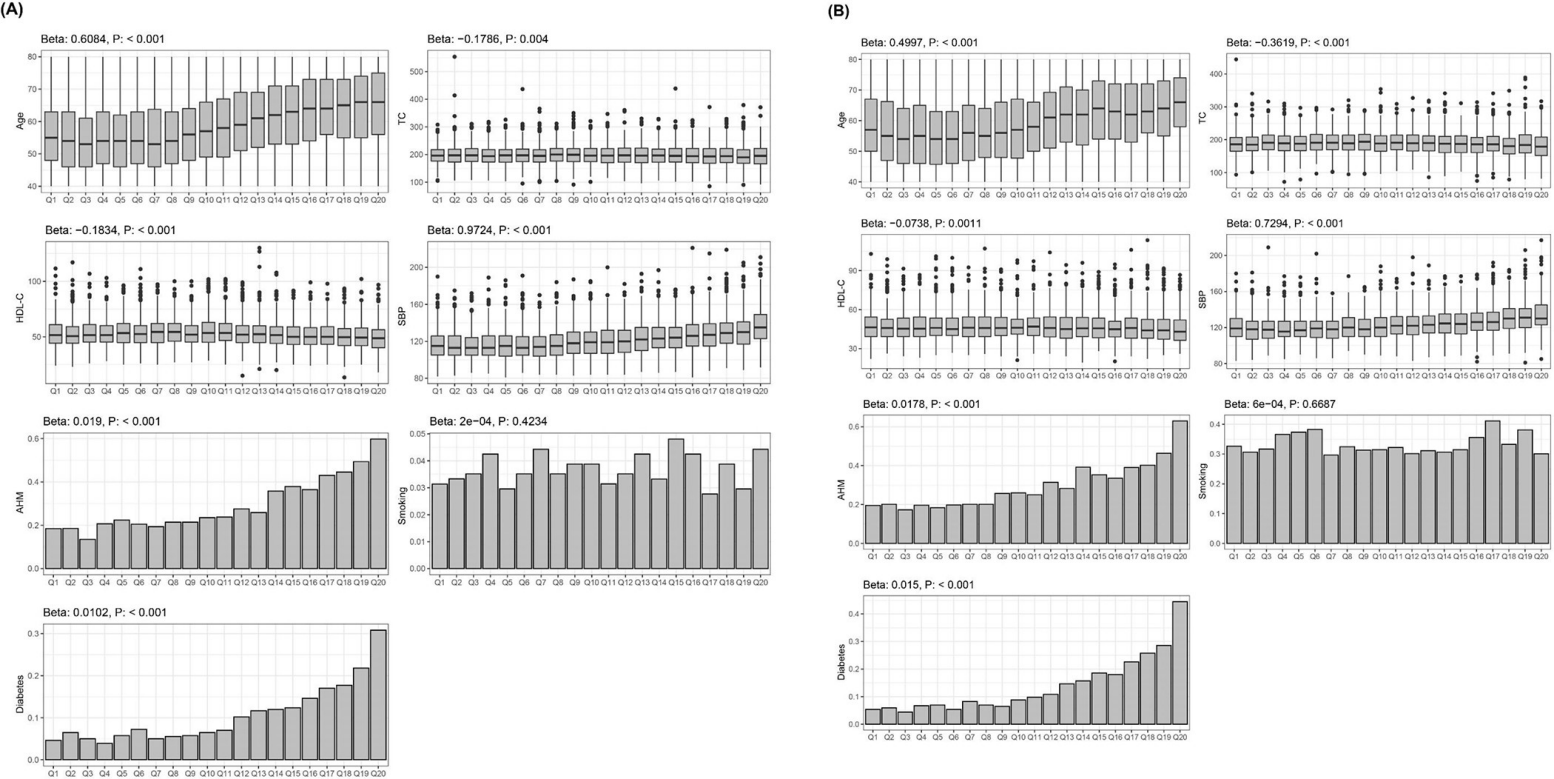
Relationship between UACR and cardiometabolic risk factors in Korean women (A) and men (B). *Beta* values were measured by linear regression after setting continuous variables, including age, TC, HDL-C, and SBP as dependent variables and UACR subgroups as independent variables. In case of features exhibiting binomial distribution, such as AHM use, smoking, and diabetes, the ratio of presence of disease or status was set as the dependent variable in the linear regression for the calculation of the *Beta* value. Abbreviations: UACR, urinary albumin-creatinine ratio; Beta, beta-coefficient; AHM, anti-hypertensive medication; HDL-C. high-density lipoprotein-cholesterol; SBP, systolic blood pressure; TC, total cholesterol.

We analyzed the association of the UACR with seven risk factors used in the calculation of FRS based on univariate analysis. In both Korean women and men, the following robust and significant signatures were associated with increase in the UACR: older age, lower TC, lower HDL-C, higher SBP, higher ratio of hypertensive medication, and higher prevalence of diabetes (Fig 2).

**Fig 3:**
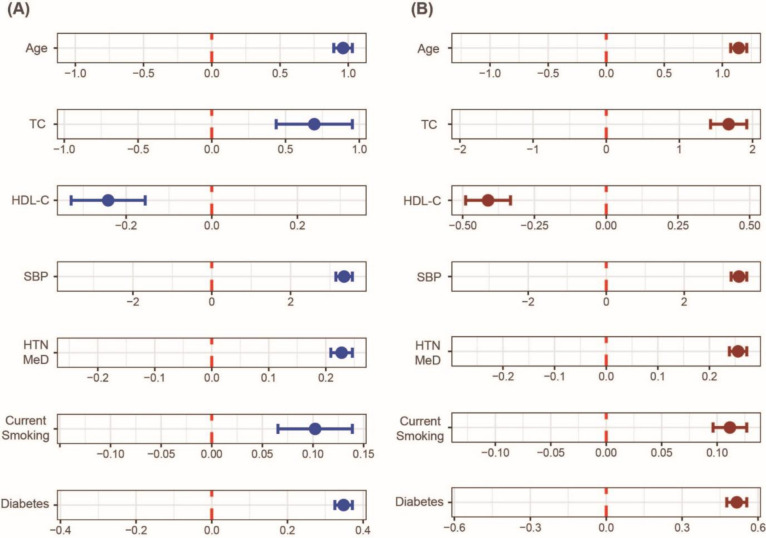
Relationship between albuminuria and cardiometabolic risk factors in Korean women (A) and men (B). Top four graphs (i.e., age, TC, HDL-C, SBP) were obtained by multivariate linear regression after setting the four predictors arranged separately as dependent variables. UACR was determined as the independent variable, and other remnant six predictors as covariates. The lower three graphs (i.e., AHM, smoking, diabetes) were obtained by multivariate logistic regression set to the same conditions as the multivariate linear regression. All x-axes indicate beta-coefficients obtained from the multivariate linear or logistic regressions. UACR levels were log-transformed for the associational analyses. Abbreviations: TC, total cholesterol; HDL-C, high-density lipoprotein cholesterol; SBP, systolic blood pressure; HTN Med, hypertension medication; DM, diabetes mellitus.

The FRS included seven cardiometabolic predictors, including age, TC, HDL-C SBP, AHM, smoking, and diabetes. Among six features, four were continuous variables set as dependent variables in each model using multivariate linear regression. The UACR was the independent variable, and the other six variables were covariates (Fig 3). In case of dichotomous variables, including AHM, smoking, and diabetes, logistic regression was used to evaluate their association with UACR after adjusting for the other remaining six predictors (Fig 3). As a result, in both Korean women and men, the high levels of UACR were related to older age, higher TC, lower HDL-C, higher SBP, greater smoking levels, greater AHM use, and diabetes were related to FRS (Fig 3).

**Fig 4:**
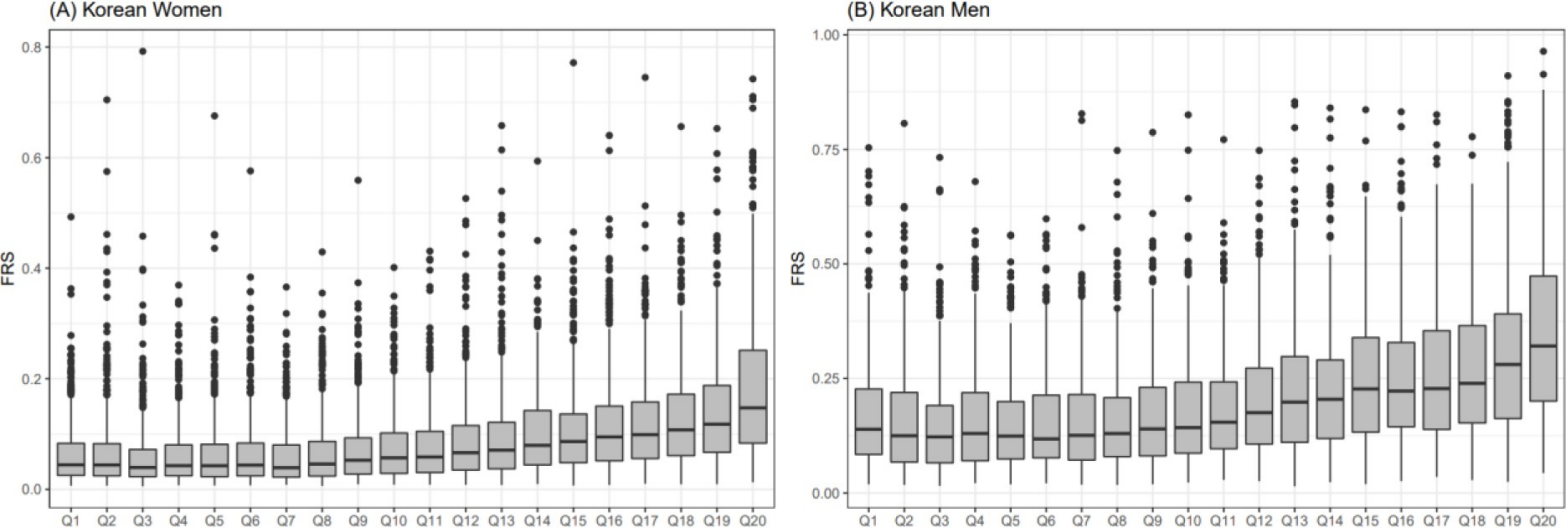
Relationship between albuminuria and FRS in Korean women (A) and men (B). Urine albumin-to-creatine ratio was categorized into 20 groups (x-axes) based on ascending order. FRS was calculated based on the equation provided by a study [22]. Abbreviation: FRS, Framingham risk score.

We compared the relationship between UACR and the combined effect of seven cardiometabolic predictors, in the form of an equation, referred to as the FRS (Fig 4). In both women and men, as UACR increased, the FRS exhibited monotonic elevated patterns. Moreover, all the increasing characteristics showed exponential distributions, indicating that the albuminuria groups (Q16 –Q20 in Fig 4) were directly proportional to extremely high risk of CVD.

## Discussion

Our study showed that both Korean women and men with CVD history had higher UACR level than those with no previous history of CVD. In particular, for those without CVD history, multivariate adjusted analysis showed that higher UACR was associated with CV risk factors such as older age, higher TC, lower HDL, higher SBP, higher proportion of HTN, higher proportion of current smoking, and higher proportion of diabetes in both Korean women and men. In both women and men without CVD history, UACR showed positive correlation with FRS. In correlation analyses between UACR and individual cardiometabolic risk factors, several non-linear correlations were shown: age, TC, HDL-C, SBP, smoking status in Korean women and men (Fig 2). Moreover, gender-specific associational findings could be observed (Fig 2). For example, in Korean men, UACR levels were negatively related to HDL-C levels (beta-coefficients: -0.1834; *p*-value < 0.001), besides, in Korean women, this trend slightly were diluted (beta-coefficient: -0.0738; p-value: 0.0011).

The HOPE and PEACE studies showed that albuminuria is associated with a higher risk of CVD incidence and mortality among high-risk patients with CVD [1, 3]. Moreover, albuminuria was associated with the risk of CVD among healthy individuals in the general population, without history of CVD [5, 26, 27]. The Multi-Ethnic Study of Atherosclerosis (MESA) study showed that UACR was associated with an 11% increase in the risk of CVD events [5]. The Framingham cohort study reported that without CVD, low level of UACR predicted the development of CVD among normotensive and nondiabetic individuals [28]. The Strong Heart Study also showed that a lower UACR than the normal value predicted CVD [27]. The Prevention of Renal and Vascular End Stage Disease Intervention Trial (PREVEND IT) study showed that the FRS is correlated with microalbuminuria [29].

The risk of CVD differs according to sex. Our results showed that the prevalence of smoking was more common among men than women, and the prevalence of obesity was higher among women than men. Moreover, women tended to have better levels of cholesterol and blood pressure. These differences have been attributed to the differences in lifestyle, health awareness, and sex hormones (such as estrogen). Recently, this difference has been decreasing; however, the difference in cholesterol and body mass index among the different sexes remains significant [30]. Because there are still differences in the control of high blood pressure, diabetes, and hyperlipidemia among different sexes, it is necessary to stratify and analyze microalbuminuria as a risk factor for CVD by sex. Our results showed that microalbuminuria could be considered an important predictor of CVD regardless of sex.

The precise pathophysiological mechanism of microalbuminuria as a CV risk factor remains unknown. The association between microalbuminuria and CVD is explained by endothelial dysfunction or chronic low-grade inflammation. Endothelial dysfunction could increase glomerular pressure and glomerular barrier permeability which increases endothelial permeability. Increased microalbuminuria could be a marker of generalized endothelial dysfunction which could predispose to an atherogenic lipoprotein accumulation in the subendothelial cell space [31, 32]. Microalbuminuria is also associated with chronic low-grade inflammation which could be both cause and consequence of endothelial dysfunction. Furthermore, endothelial dysfunction and low-grade inflammation can not only lead to atherothrombosis but can also be independently associated as a risk for CVD [32, 33].

Diabetic patients have an increased risk of microalbuminuria and 20–30% of patients with diagnosed diabetes have been found to have microalbuminuria [34]. Those patients have abnormal insulin resistance and increased serum glucose level that makes serum insulin level increase. Insulin stimulates store-operated Ca entry via Orai-1 channel in podocytes that makes actin remodeling and transepithelial albumin leakage resulting in microalbuminuria [35]. Recently over 8 years follow up study from Korean Genome and Epidemiology Study (KOGES) showed that microalbuminuria could be used as an early marker of progression to diabetes even in the non-prediabetic population [36]. Through microalbuminuria we could predict abnormal insulin resistance and diabetes, which were major risk factors for cardiovascular disease [37, 38].

This study has several limitations. First, this was a cross-sectional designed study, therefore, a causal relationship between the exposure (i.e., UACR) and outcome (i.e., CVD status) could not be established, but only association between them could only be identified. To establish causation between UACR and CVD status, a longitudinal study design, intervention study design, or study using mendelian randomization analysis [39] is required. Second, we implemented the FRS to categorize subjects into binomial groups, including high- and low-risk CVD groups. Two reasons not to use the real CVD status obtained from a questionnaire for individuals’ current condition or diagnosis with CVD: the prevalence of CVD in KNHANES is extremely low, therefore, could yield the biased results; the real CVD status only reflects a subject’s current condition, besides, the FRS could predict their future risk of the incidence of CVD. Third, we could not consider the use of medications such as angiotensin-converting enzyme inhibitors or angiotensin receptor blockers which may reduce the degree of microalbuminuria. Fourth, we used a single urine spot sample to assess the UACR rather than the 24-hour urine collection or multiple samples. Nevertheless, we investigated the correlation between microalbuminuria and CVD in a single representative group by using nationally notarized data.

In conclusion, our study showed that UACR level was associated with FRS in both women and men with no previous history of CVD. In addition to FRS, measuring UACR is a cost-effective tool for predicting and preventing CVD in both sexes.

## Supporting information

S1 Fig Distribution of urine albumin (upper two figures), urinary creatinine (middle figure), and urinary albumin creatinine ratio (lower two figures) in Korean women.
(DOCX)

S2 Fig Distribution of urine albumin (upper two figures), urinary creatinine (middle figure), and urinary albumin creatinine ratio (lower two figures) in Korean men.
(DOCX)
