# Supplementary figures and images for: Association between urinary albumin creatinine ratio and cardiovascular disease
Source: PLoS One. 2023 Mar 21;18(3):e0283083. doi: 10.1371/journal.pone.0283083 (PMC10030008; doi:10.1371/journal.pone.0283083)

**
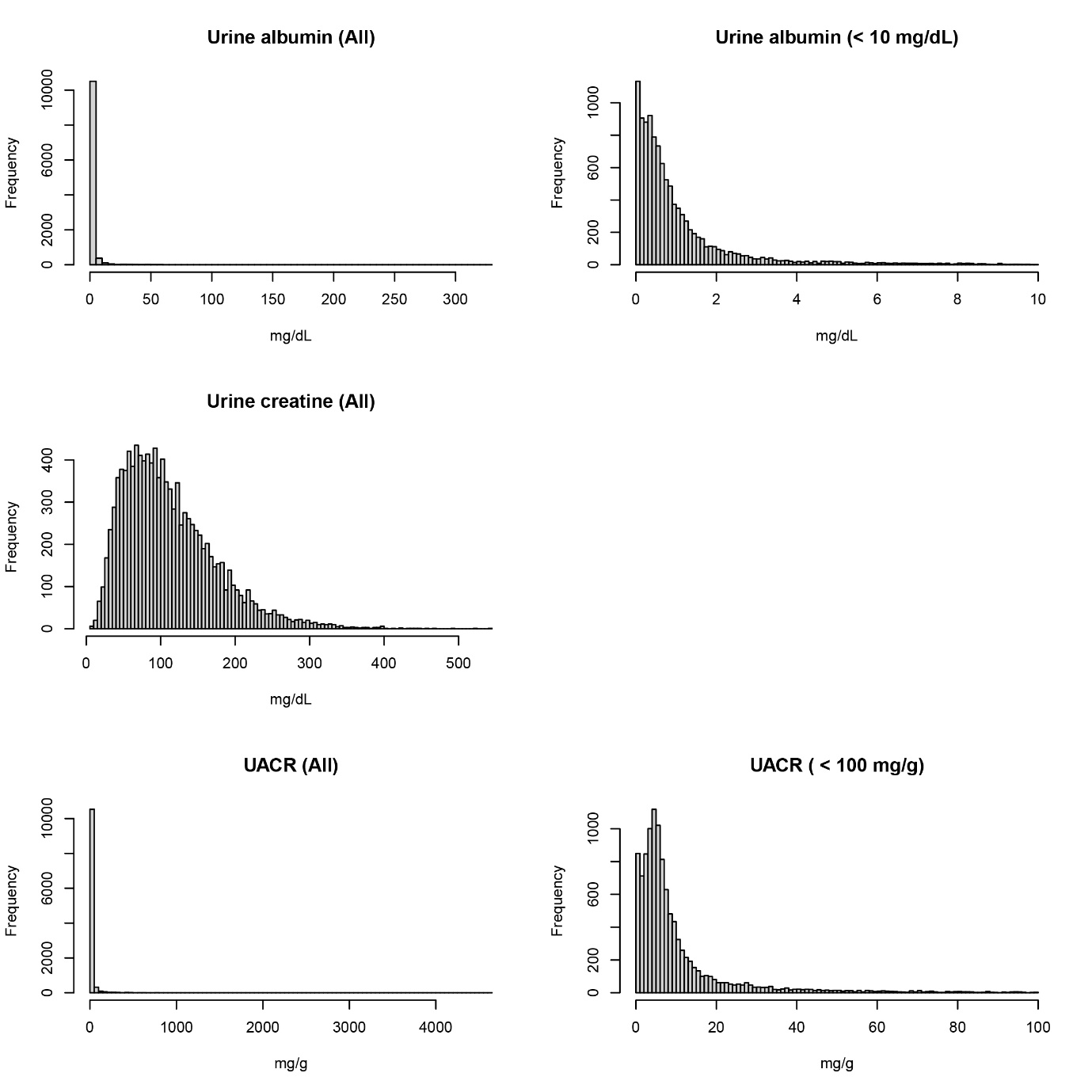
**

Supplement: S1 Fig — Distribution of urine albumin (upper two figures), urinary creatinine (middle figure), and urinary albumin creatinine ratio (lower two figures) in Korean women. (DOCX) [file pone.0283083.s001.docx]

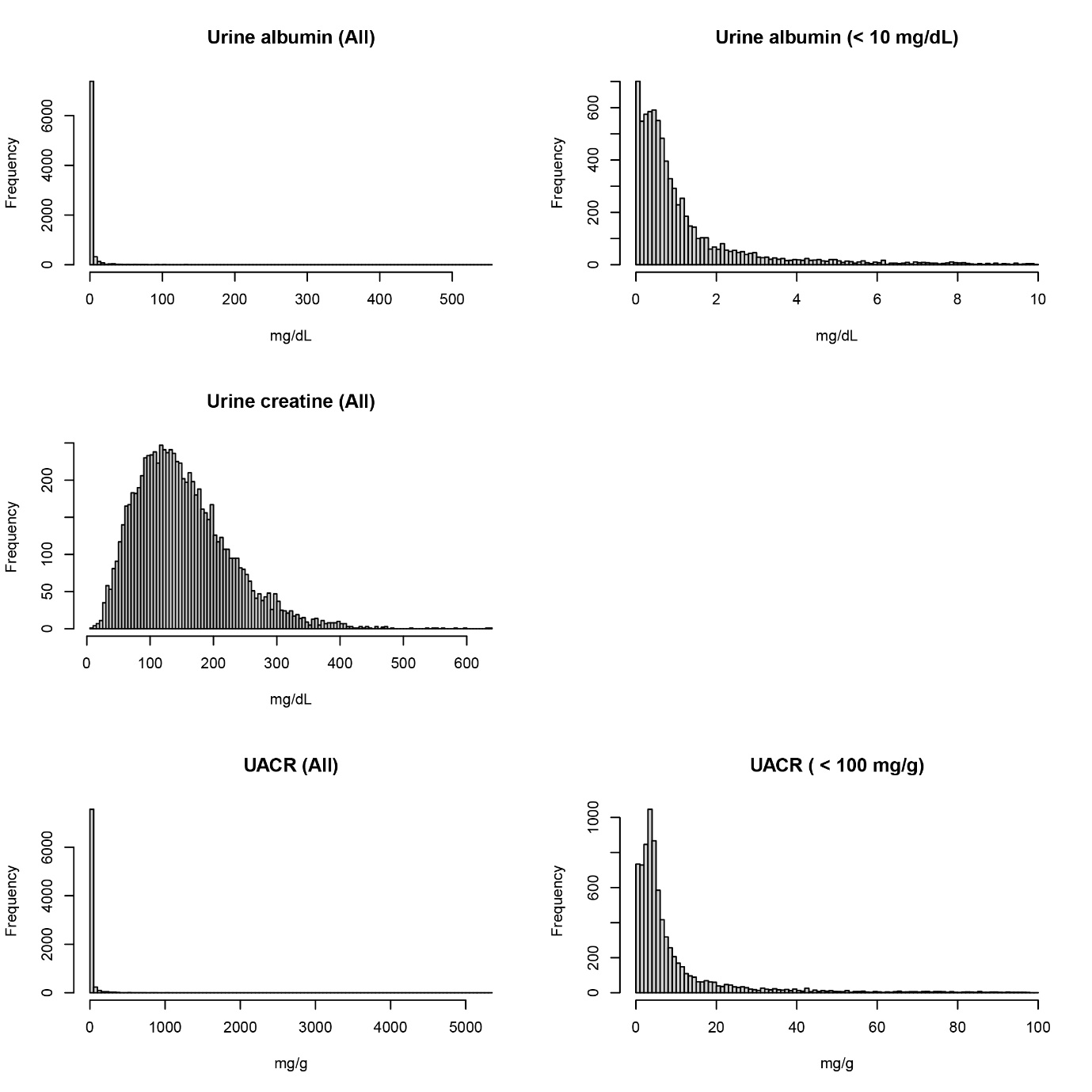

Supplement: S2 Fig — Distribution of urine albumin (upper two figures), urinary creatinine (middle figure), and urinary albumin creatinine ratio (lower two figures) in Korean men. (DOCX) [file pone.0283083.s002.docx]
